# Supplementary material for: Splice-Junction-Based Mapping of Alternative Isoforms in the Human Proteome
Source: Cell Rep. Author manuscript; Available in PMC 2020 Jan 15. (PMC6961840; doi:10.1016/j.celrep.2019.11.026)
Supplement: 2 [file NIHMS1546469-supplement-2.zip › DF1/PXD010154/Lung/Lung_19_YWHAZ_GDEAEAGEGGERFGVSYPGIVDQSQQAYQEAFEISK.pdf]

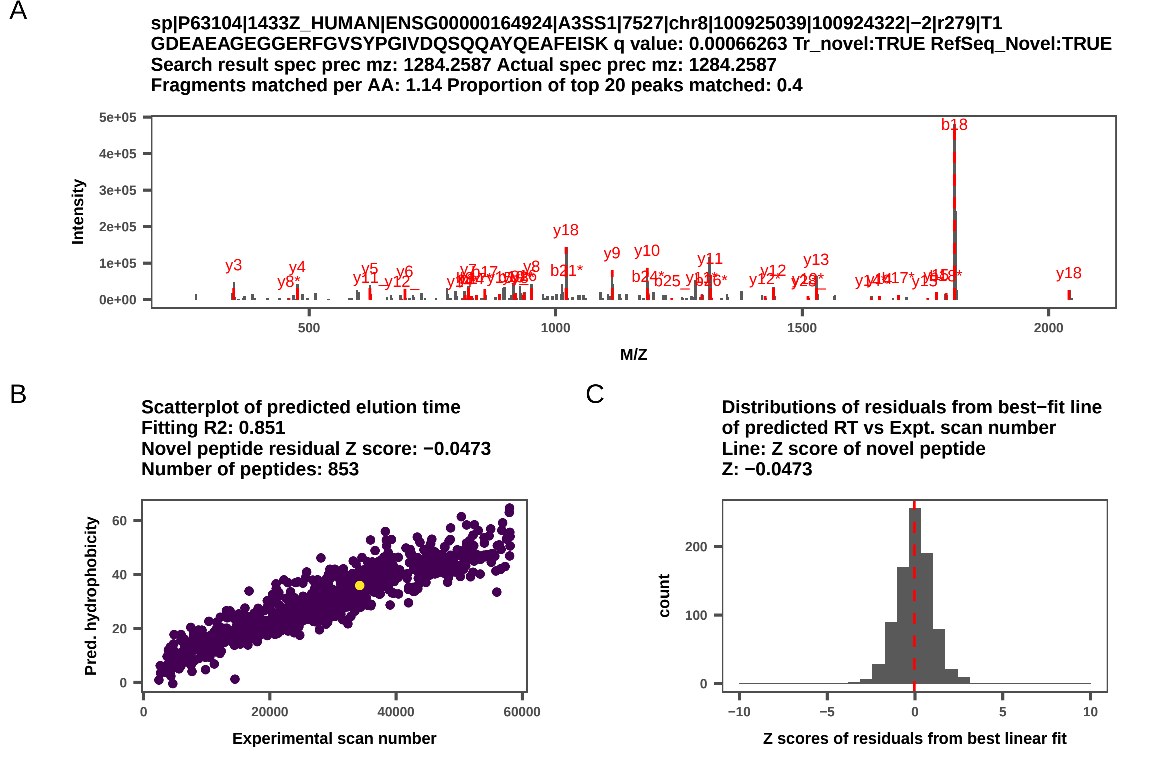
A
sp|P63104|1433Z_HUMAN|ENSG00000164924|A3SS1|7527|chr8|100925039|100924322|−2|r279|T1
GDEAEAGEGGERFGVSYPGIVDQSQQAYQEAFEISK q value: 0.00066263 Tr_novel:TRUE RefSeq_Novel:TRUE
Search result spec prec mz: 1284.2587 Actual spec prec mz: 1284.2587
Fragments matched per AA: 1.14 Proportion of top 20 peaks matched: 0.4
Intensity
5e+05
4e+05
3e+05
2e+05
1e+05
0e+00
M/Z
500
1000
1500
2000
b18
y18
y3
y4
y8*
y5
y11
y6
y7
y8
b21*
y9
y10
b24*
y11
y12
y12*
y13
y15
y18
B
Scatterplot of predicted elution time
Fitting R2: 0.851
Novel peptide residual Z score: −0.0473
Number of peptides: 853
Pred. hydrophobicity
60
40
20
0
0
20000
40000
60000
Experimental scan number
C
Distributions of residuals from best−fit line
of predicted RT vs Expt. scan number
Line: Z score of novel peptide
Z: −0.0473
count
200
100
0
−10
−5
0
5
10
Z scores of residuals from best linear fit
